# TRPC Channels Activated by G Protein-Coupled Receptors Drive Ca^2+^ Dysregulation Leading to Secondary Brain Injury in the Mouse Model

**DOI:** 10.1007/s12975-023-01173-1

**Published:** 2023-07-18

**Authors:** Jasneet Parmar, Georg von Jonquieres, Nagarajesh Gorlamandala, Brandon Chung, Amanda J. Craig, Jeremy L. Pinyon, Lutz Birnbaumer, Matthias Klugmann, Andrew J. Moorhouse, John M. Power, Gary D. Housley

**Affiliations:** 1 Translational Neuroscience Facility and Department of Physiology, School of Biomedical Sciences, UNSW Sydney, Sydney, NSW 2052 Australia; 2 Institute of Biomedical Research (BIOMED), Pontifical Catholic University of Argentina, Av. A Moreau de Justo 1300, C1107AFF Buenos Aires CABA, Argentina; 3 Laboratory of Signal Transduction, National Institute of Environmental Health Sciences, Research Triangle Park, North Carolina 27709 USA

**Keywords:** *Trpc* knockout mouse models (*Trpc3*, *Trpc1/3/6/7*, *GAD67-GFP-Trpc3*^*KO*^), AAVrh20-GCaMP5g genetically encoded Ca^2+^ reporter, Photothrombotic focal ischemic brain injury model, Neuroprotection, Glutamate excitotoxicity

## Abstract

Canonical transient receptor potential (TRPC) non-selective cation channels, particularly those assembled with TRPC3, TRPC6, and TRPC7 subunits, are coupled to G_αq_-type G protein-coupled receptors for the major classes of excitatory neurotransmitters. Sustained activation of this TRPC channel-based pathophysiological signaling hub in neurons and glia likely contributes to prodigious excitotoxicity-driven secondary brain injury expansion. This was investigated in mouse models with selective *Trpc* gene knockout (KO). In adult cerebellar brain slices, application of glutamate and the class I metabotropic glutamate receptor agonist (S)-3,5-dihydroxyphenylglycine to Purkinje neurons expressing the GCaMP5g Ca^2+^ reporter demonstrated that the majority of the Ca^2+^ loading in the molecular layer dendritic arbors was attributable to the TRPC3 effector channels (*Trpc3*^*KO*^ compared with wildtype (WT)). This Ca^2+^ dysregulation was associated with glutamate excitotoxicity causing progressive disruption of the Purkinje cell dendrites (significantly abated in a *GAD67-GFP*-*Trpc3*^*KO*^ reporter brain slice model). Contribution of the G_αq_-coupled TRPC channels to secondary brain injury was evaluated in a dual photothrombotic focal ischemic injury model targeting cerebellar and cerebral cortex regions, comparing day 4 post-injury in WT mice, *Trpc3*^*KO*^, and *Trpc1/3/6/7* quadruple knockout (*Trpc*^*QKO*^), with immediate 2-h (primary) brain injury. Neuroprotection to secondary brain injury was afforded in both brain regions by *Trpc3*^*KO*^ and *Trpc*^*QKO*^ models, with the *Trpc*^*QKO*^ showing greatest neuroprotection. These findings demonstrate the contribution of the G_αq_-coupled TRPC effector mechanism to excitotoxicity-based secondary brain injury expansion, which is a primary driver for mortality and morbidity in stroke, traumatic brain injury, and epilepsy.

**Supplementary Information:**

The online version contains supplementary material available at 10.1007/s12975-023-01173-1.

## Introduction

Pathophysiological dysregulation of extracellular glutamate and other excitatory neurochemicals drives secondary expansion of brain injury in ischemic stroke, traumatic brain injury (TBI), and epileptiform excitotoxicity. Collectively, these neuropathological conditions lacking effective treatment options represent a major unmet clinical need as leading causes of death and profound morbidity. Following initial brain injury, the on-going pathophysiological sequelae of neurotransmitter dysregulation, oxygen-glucose deprivation, innate immunoreactive activation, and run-down of cell metabolism and ion transporters, notably Na^+^/K^+^-ATPase, plasma membrane Ca^2+^-ATPase, and Na^+^/Ca^2+^ exchanger, are tied to sustained membrane depolarization and Ca^2+^ dysregulation. This drives irreversible Ca^2+^-dependent enzyme activation triggering necrosis, apoptosis, and autophagy in neurons and glia [1–3]. Targeting these mechanisms to achieve neuroprotection has been a fraught translational focus [3–5]. While Ca^2+^ entry via canonical transient receptor potential (TRPC) ion channels has been implicated in secondary brain injury [6–8], the degree to which recruitment of these channels through excitatory neurotransmitter coupling serves to drive Ca^2+^ overload underlying secondary brain injury expansion has not been established, but likely reflects a confluence point of pathophysiological signaling in the hours and days following the acute insult.

TRPC ion channels are non-selective cation channels assembled as tetramers of subunits (TRPC1–7) in homo- and/or heteromeric configurations with differing conductance properties and polymodal activation mechanisms, reflecting diversity in signal transduction throughout the body [9–11]. Expression is prominent in the CNS, where the TRPC1 subunit can co-assemble with TRPC4 and TRPC5 subunits [12], as well as with the TRPC3, TRPC6, and TRPC7 subunits to confer sensitivity to the G_αq_-type G protein-coupled receptor (GPCR) signal transduction pathway [11, 13]. Activation of G_αq_-GPCRs triggers phospholipase C_β_ (PLC)-mediated conversion of phosphatidylinositol 4,5-bisphosphate (PIP_2_) into inositol trisphosphate (IP_3_) and diacyglycerol (DAG), with DAG readily activating channels incorporating TRPC3, TRPC6, and TRPC7 subunits (Fig. S1). TRPC channels are co-expressed with class I (G_αq_-coupled) metabotropic glutamate receptors (mGluR) in neurons, astrocytes, microglia, oligodendroctyes, and endothelial cells of the microvasculature [14–17] and are associated with homeostasis of the neurovascular unit, glia-neuron communication, innate immune status, and regulation of the blood brain barrier. This diverse functionality broadly ties TRPC channels into the pathophysiology of brain injury [6, 8, 16, 18, 19].

The extensive distribution of *Trpc* subunit gene expression in the CNS has largely been determined by in situ hybridization localization of mRNA complemented by single cell transcriptomic profiling in mouse, non-human primate, and human brain tissue [20]. The latter database indicates that across species *Trpc* channel expression is evident in all grey matter regions. In cortex and hippocampus, single-neuron mRNA transcript expression is ranked as *Trpc1>Trpc5>Trpc6>Trpc4>Trpc3>Trpc7* (*Trpc2* is a pseudogene in humans and has minimal representation in the mouse CNS), co-incident with group I mGluR (*Grm1* and *Grm5* expression). The TRPC4/5 subclass of channels has recently been shown to contribute to ischemic brain injury, where use of knockout (KO) mouse models, particularly *Trpc4*^*KO*^, showed neuroprotection in the medial cerebral artery occlusion (MCAO) stroke model [8]. Study of hippocampal excitotoxicity in a mouse epilepsy model indicated significant contributions from *Trpc1/4/5* [21, 22]. Regional and neuron-specific differences in *Trpc* gene expression are particularly notable with regard to GPCR-G_αq/11_-coupled TRPC effectors, for instance, *Trpc3* expression is maximal in cerebellar Purkinje neurons, and in the hippocampus region, the dentate gyrus and pyramidal cells [23]. In comparison, *Trpc6* mRNA transcript levels are highest in the dentate gyrus of the hippocampus and cortex, and moderate in the cerebellar granule cell region [2], reflecting functional differentiation.

In mouse cerebellar Purkinje neurons, homomeric TRPC3 channels are the sole effector of the class I metabotropic glutamate receptor (mGluR1) and generate the slow excitatory post-synaptic currents (sEPSC) [24], which are absent in *Trpc3* knockout mice (*Trpc3*^*KO*^). Analysis of the *Trpc3* mRNA transcript isoforms from the cerebellum in mouse, rat, guinea pig, and human indicates consistent regulation of alternative splicing to favor production of the *Trpc3c* isoform which lacks exon 9, encoding the proximal part of the cytosolic calmodulin-IP_3_R binding (CIRB) domain coding region [6, 25]. A consequence of this splicing is that Purkinje neurons express a TRPC3 homomeric channel with an enhanced Ca^2+^ conductance lacking Ca^2+^ feedback inhibition, coupled to the mGluR1 GPCR [6]. Despite having a primary mode of activation linked to neurotransmitter release, and with a Ca^2+^ conductance comparable to those of the NMDA-type ionotropic glutamate receptor, relatively little attention has been paid to the potential of these channels to contribute to brain injury.

A study of the *Trpc3/6/7* triple KO mice in the MCAO ischemic reperfusion stroke model indicated significant reduction in infarct volume [26] and implicated astrocytic apoptosis in the *Trpc*-mediated brain injury process. This was supported by an in vitro study of reactive astrogliosis in culture which showed that astrocytes lacking *Trpc1* expression but retaining *Trpc3* expression had heightened Ca^2+^ entry [14]. With regard to ischemic brain injury, while NMDA ionotropic glutamate receptor-mediated Ca^2+^ dysregulation with sustained glutamate release is considered the principal driver of excitotoxicity [3, 5, 27, 28], the group I mGluR (mGluR1/5) is strongly implicated in ischemic brain injury [29, 30].

Here we evaluate the contribution of the mGluR-coupled TRPC effector channels to this pathophysiological process, utilizing *Trpc3* (*Trpc3*^*KO*^), *Trpc1/3/6/7* (*Trpc*^*QKO*^), and *GAD67-GFP-Trpc3*^*KO*^ mouse models to determine TRPC channel-mediated Ca^2+^ dysregulation and glutamatergic excitotoxicity in cerebellar Purkinje neurons, and how mGluR-TRPC channel coupling contributes broadly to progressive secondary brain injury in cerebellar and cerebral focal ischemic stroke.

## Materials and Methods

### Animals

All animal procedures were conducted in accordance with the Australian Code of Practice for the Care and Use of Animals for Scientific Purposes (National Health and Medical Research Council) and were approved by the UNSW Sydney Animal Care and Ethics Committee. Transgenic mouse lines used in this study were *Trpc3*^*KO*^, *Trpc1,3,6,7* quadruple KO (*TRPC*^*QKO*^), *GAD67-GFP* knockin (*GAD67-GFP*^*+*^), and *GAD67-GFP*^*+*^*-Trpc3*^*KO*^. These mouse lines were on a mixed C57Bl/6J/129-SvEv background. The breeding and housing were undertaken at the Australian BioResources Facility (Moss Vale, Sydney), from founding *Trpc* transgenic mouse lines provided by L.B. via National Institute of Environmental Health Sciences, Research Triangle Park, NC, USA., and a *GAD67-GFP*^*+*^ mouse line was provided by Prof. Yuchio Yanagawa, Gunma University, Japan [31]. All experiments utilized gender- and age-matched WT (C57BL/6J/129-SvEv) mice. The mice were of both genders and maintained on a 12-h light/dark cycle and fed ad libitum.

### AAV Delivery of Genetically Encoded Ca^2+^ Reporter into the Neonatal Mouse Brain

A recombinant adeno-associated virus (AAVrh20) driving GCaMP5g expression under the control of the 1.1-kb CMV enhancer/chicken beta-actin promotor (CAG) custom produced in the UNSW Translational Neuroscience Facility was injected into the cerebellae of WT and *TRPC3*^*KO*^ mouse pups (postnatal day 3). Pups were cryo-anesthetized for 5 min on ice followed by free-hand injection of the AAV (2 mm posterior to the mid-auricle line) in the cerebellum (2 μl of 2 × 10^12^ vg/ml titer). Following the injection, the pups were kept on a heat pad (37 °C) to rewarm and then returned to the dam and monitored every hour for the first 3, and then twice every day for 4 weeks.

### Acute Cerebellar Brain Slice Preparation for Ca^2+^ Imaging

AAV-injected mice at 6–8 weeks of age were euthanized with pentobarbital (Virbac, Sydney, Australia; intraperitoneal (i.p.) administration). The mice were decapitated, and whole brains dissected and immersed in ice-cold, modified high-sucrose artificial cerebrospinal fluid (aCSF) consisting of (in mM): 246 sucrose, 26 NaHCO_3_, 10 glucose, 4 KCl, 5 MgCl_2_, and 1 CaCl_2_. The cerebellum was separated from the rest of the brain, and its lateral side affixed to the vibratome (VT 1200, Leica, Germany) cutting platform with cyanoacrylate adhesive. The cutting platform was placed in the holding chamber of the vibratome which contained the high sucrose, modified aCSF solution with constant carbogen (95% O_2_ and 5% CO_2_) aeration. Parasagittal slices (400 μm) were obtained and transferred to a slice holding chamber containing standard aCSF solution (in mM): 119 NaCl, 26.2 NaHCO_3_, 11 glucose, 2.5 KCl, 1 NaH_2_PO_4_, 2.5 CaCl_2_, and 1.3 MgCl_2_, continuously aerated with carbogen. Slices were incubated for a minimum of 1 h in standard aCSF at room temperature prior to imaging.

### Confocal Imaging of Acute Brain Slices Using the GCaMP5g Ca^2+^ Reporter

All imaging was performed at room temperature using the Zeiss confocal laser scanning microscope system (710NLO LSM) with 488-nm excitation wavelength to excite the GCaMP5g Ca^2+^ reporter protein, and the emission capture was kept constant at 493–598 nm. Slices were transferred to a bath setup on the confocal microscope stage and held in place by a nylon mesh to restrict movement. The slices were continuously superfused with carbogen-bubbled aCSF at a flow rate of 2 ml/min, and all drugs were bath applied in aCSF. TPC-channel-mediated Ca^2+^ entry was activated by bath superfusion with glutamate (4 mM), or the class I mGluR selective agonist (S)-3,5-Dihydroxyphenylglycine (DHPG; 100 μM). The laser power and detector gain were adjusted for each slice to achieve a good contrast and sensitivity. WT slices were excited with 3–33% laser power, master gain for the PMT detector was 720–905, and digital gain was 1–1.5. *Trpc3*^*KO*^ slices were excited with 11–50% laser power, master gain 800–875, and digital gain 1–2. The pixel dwell time was 3.15 μs for all experiments. Images were captured every 10 s in 8-bit resolution.

Image analysis was performed using Image J software (NIH). A region of interest (ROI) was first drawn around the entire image to obtain mean intensity for the whole field. To analyze fluorescence responses from a single Purkinje neuron, an ROI was drawn around the Purkinje neuron soma using specific criteria. If somata from two Purkinje neurons overlapped, the neurons were not included in the analysis. Furthermore, if the neurons did not recover and showed sustained elevation of the GCaMP5g fluorescence signal during the drug washout period, they were considered damaged and excluded from the dataset. Slices from the same animal were treated as separate samples. Responses from individual Purkinje neurons within a slice were averaged to represent one data point. All data were reported as ΔF/F_0_, where F_0_ is the average of three baseline fluorescence values immediately following the addition of the drug, and ΔF is the difference between a fluorescence value and F_0_.

### Acute Cerebellar Brain Slice Preparation for Assessing Dendritic Damage

Acute brain slice preparations of the cerebellum (parasagittal orientation, 400-μm thickness) were performed as described above from *GAD67-GFP*^*+*^ and *GAD67-GFP*^*+*^*-Trpc3*^*KO*^ mice (males and females, aged 6–8 weeks; blinded to genotype) and incubated for 1 h in standard aCSF at 31 °C to allow for recovery. One slice from both *GAD67-GFP*^*+*^ and *GAD67-GFP*^*+*^*-Trpc3*^*KO*^ mice was fixed immediately after the end of 1-h recovery period as control slice (*t* = 0) while another slice was incubated with 1-mM glutamate in aCSF for 1 h and then fixed. Fixation was performed overnight in 4% paraformaldehyde (PFA) (Sigma-Aldrich, Germany) in aCSF, at 4 °C, then stored in 0.01 M phosphate buffered saline (PBS) (Sigma-Aldrich, USA) at 4 °C until imaging.

### Imaging of the *GAD67-GFP*^*+*^ Cerebellar Brain Slices

Three-dimensional (3D) multiphoton imaging reconstructions of four randomly selected ROIs within cerebellar brain slices were generated by compiling a series of *z*-stack images. Stacks were captured using 20× objective (1.2 NA)/1.5× zoom at 2-μm *z*-intervals using a Ti: sapphire Mai Tai DeepSee pulsed infrared laser (Spectra-Physics, California, USA; excitation 875 nm, emission 500–545 nm). The thickness of the composite imaged slices ranged from 50 to 150 μm, set using the appearance of first and last Purkinje neuron soma. Images were analyzed using the ImageJ software (NIH) to measure the length of the Purkinje neuron primary dendrites in the molecular layer (ML). The primary dendrite projection was calculated as a percentage of the ML width at that point in the slice. Data from 3 to 4 Purkinje neurons per ROI from four randomly selected ROIs in each slice was averaged to represent one data point. The Purkinje neuron soma surface area was determined using a semi-automated algorithm using the “autoNeuron” module of the Neurolucida software (11.03, MBF Bioscience - MicroBrightField, Inc., USA). A ROI was demarcated manually, and the program traced the somas three-dimensionally by tracing the outline of each soma through all relevant z-stacks.

### Induction of Dual Photothrombotic Brain Lesions

To assess the impact of *Trpc* gene deletion on secondary brain injury expansion arising from focal ischemia in the cerebrum and cerebellum, photothrombotic lesions [32, 33] were delivered in both these regions simultaneously in age-matched WT, *Trpc3*^*KO*^, and *Trpc*^*QKO*^ mice (male and female mice aged 7–14 weeks; blinded for genotype; Table S1 for gender and age distribution). Anesthesia was induced with 3% isoflurane (balanced with O_2_), mice were fixed onto a stereotaxic frame (Kopf Instruments, US), and the anesthesia was maintained at 1.5% isoflurane for the duration of the procedure. Body temperature was maintained at 37 ± 0.5 °C, and the heart rate and O_2_ saturation were constantly measured throughout surgery (PhysioSuite®, Kent Scientific, US). An ophthalmological gel (Alcon Laboratories Ltd, New Zealand) was applied to the eyes for corneal protection. Temgesic (Buprenorphine; 0.15 mg/kg; 0.32 mg/ml) was administered intramuscularly (i.m.), using a 27G needle.

The head was shaved, and local anesthetic lignocaine was injected subcutaneously (27 needle; 20 mg/ml) over the skull. A midline incision was made through the skin to expose the cranium and the periosteum removed, followed by application of the ophthalmic gel (Alcon Laboratories Ltd, New Zealand) onto the surface of the skull to improve translucency. Two optical fiber-coupled green LEDs (1-mm diameter aperture, 0.5 NA (M59L01), 530-nm LED (M530F2); 1 mW—calibrated using a digital fiber power meter (PM20A), Thorlabs Inc.) were mounted on a stereotaxic frame to target the cortex (2 mm anterior; 3 mm left from the lambda point) and cerebellum (2 mm posterior from the lambda point, at a 30 ° angle), and advanced to make contact with the surface of the skull.

Once the two optical fibers were correctly positioned, the animals were injected with the photosensitizing rose Bengal dye (50 mg/kg; 1 ml/kg, Sigma) via the tail vein (29G needle), following which the green LED illumination was applied for 5 min to trigger localized thrombus formation. The skin was sutured, and the isoflurane anesthesia removed. Mice were hydrated with normal saline (i.p., 250 μl) during the recovery phase and maintained on the rectal probe feedback-controlled heating pad in a recovery cage until ambulatory and then placed back in their home cage with adequate food and water. Brain tissue collection was performed at 2-h post-injury (hpi) from WT mice to calculate primary injury size (reference), and at 4-day post-injury (dpi) from WT, *Trpc3*^*KO*^, and *Trpc*^*QKO*^ mice, to assess secondary injury expansion. No mortality was observed during the procedure, and one animal in the WT group died within 12 h. Necropsy showed significant clotting in the brainstem.

### Infarct Quantification Using Brain Surface Infarct Perimeter Mapping and Cryosection Darkfield Imaging

Mice were euthanized with pentobarbital (i.p.; Virbac, Sydney, Australia;) following which they were transcardially perfused with 4% paraformaldehyde (PFA). The brains were dissected and post-fixed overnight in 4% PFA at 4 °C. The brains were then cryoprotected in 10% and 30% sucrose in 0.01 M PBS solutions overnight. Images of the surface infarcts were obtained for perimeter mapping of infarct extent using a stereomicroscope (M80, Leica; 0.8× objective, 1.25× internal zoom; attached camera, IC80HD, Leica). The brains were serially cryosectioned (coronal; 50 μm thick) using a cryostat (Leica) and collected in 0.01 M PBS. Unstained, free floating cryosections (in PBS) were imaged using a stereomicroscope (Leica M80, 0.8× objective; 1.25× internal zoom; attached camera IC80HD, Leica) with auxiliary LED light guides (XL200, Leica) to achieve differential darkfield illumination of the tissue. The darkfield images were converted to greyscale in Photoshop software (Adobe).

Image J software (NIH) was used to demarcate the cross-sectional areas of infarcts. This included surface infarct cross-sectional areas determined from images of the intact brain, as well as measurements of cross-sectional areas of serial sections under darkfield imaging. Infarct volumes were quantified by summation of cross-sectional infarct areas and integrated with the thickness of the sections (50 μm). The assessment of infarct was performed blinded to the genotype. Following data collection, the genotypes were revealed to perform data analysis.

### Statistics

Data were tested for normality and analyzed using ANOVA or *t* test using the Sigmaplot software (Systat, USA). Multiple pairwise comparisons were conducted using the Holm-Sidak method, and data are represented as mean ± standard error of mean (SEM). If data were not normally distributed, analysis was performed on ranked data.

## Results

### Ca^2+^ Loading via the mGluR1-TRPC3 Pathway in Cerebellar Purkinje Neurons

The contribution of the mGluR1–TRPC3 ion channel Ca^2+^ entry pathway to glutamatergic receptor-mediated Ca^2+^ loading with sustained activation (modelling excitotoxicity) was assessed by recording GCaMP5g-based Ca^2+^ fluorescence in WT and *Trpc3*^*KO*^ adult mouse cerebellar brain slices using confocal Ca^2+^ imaging. The brain slices were superfused with aCSF, aCSF + glutamate (4 mM), or the mGluR1 agonist DHPG (100 μM), with washout in aCSF; WT and *Trpc3*^*KO*^ slices were interspersed. These experiments selectively resolved Ca^2+^ signaling in Purkinje neurons, as granule cell neurons did not express GCaMP5g (Fig. S2).

**Fig. 1:**
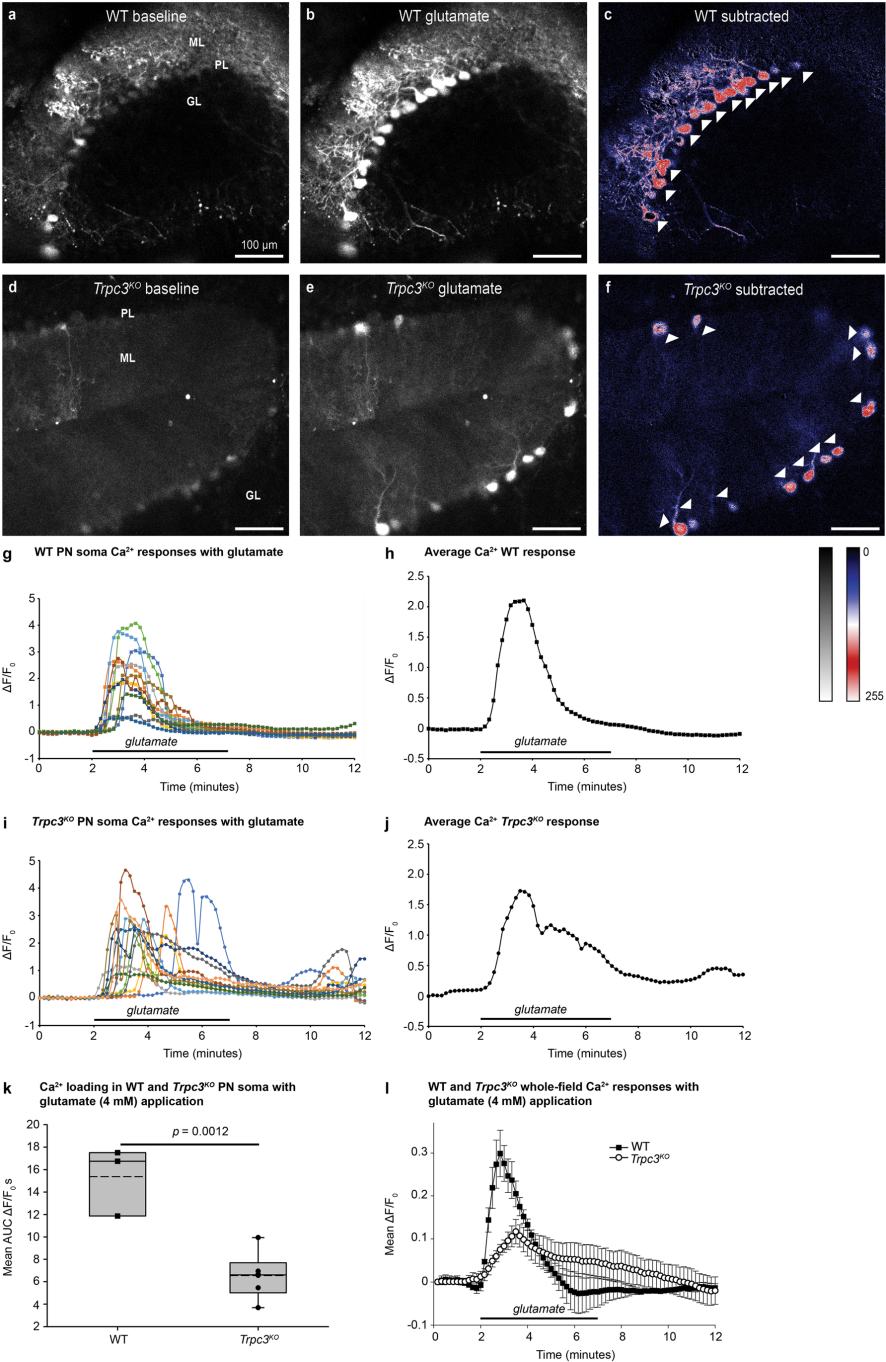
Ca^2+^ loading in Purkinje neurons evoked by application of glutamate from WT or *Trpc3*^*KO*^ adult mouse cerebellar brain slices following AAV-GCaMP5g–transfection at P3. **a** WT baseline indicating the cerebellar Purkinje neuron layer (PL), the dendritic arbors (molecular layer, ML), and granule cell layer (GL). **b** Peak Ca^2+^ fluorescence with 4-mM glutamate stimulation. **c** Glutamate-mediated Ca^2+^ loading demonstrated by subtraction of the baseline image prior to glutamate application (**b**−**a**). In comparison, Ca^2+^ loading in the *Trpc3*^*KO*^ tissue (**d**–**f**) was largely confined to the PL, putatively representing AMPA-type glutamate receptor-mediated Ca^2+^ entry and Ca^2+^ store release. Furthermore, the lack of Ca^2+^ loading in the ML with glutamate application in *Trpc3*^*KO*^ tissue indicates the dominance of this Ca^2+^ entry pathway in this region. **d** Baseline. **e** Peak Ca^2+^ with glutamate application. **f** Ca^2+^ loading subtracted image (**e**−**d**). **g**, **h** WT individual and averaged Purkinje neuron soma Ca^2+^ signals in a representative brain slice. Adaptation occurs within the glutamate superfusion period. **i**, **j** *Trpc3*^*KO*^ individual and average Purkinje neuron soma responses from a representative brain slice. Adaptation to baseline during glutamate superfusion is comparable to WT. **k** Comparison of the average area under the curve (AUC) for Purkinje neuron somata responses for the initial 2 min of glutamate application, across brain slices for WT (*n* = 3) and *Trpc3*^*KO*^ (*n* = 6). Box plots reflect 25% and 75% quartiles, with data overlay. Dashed lines show mean values; solid lines show the median. (*p* = 0.0012, unpaired *t* test). **l** Comparison of glutamate-induced change in Ca^2+^ fluorescence intensity for the whole field of view of WT and *Trpc3*^*KO*^ brain slices (reflecting both PN soma and dendritic Ca^2+^ signal). The differential in peak responses reflects the significance of Ca^2+^ entry via the mGluR1-TRPC3 pathway. Data shown as mean ± SEM. Ca^2+^ fluorescence following AAV-GCaMP5g PN transfection to these adult mice at post-natal day 3

In WT brain slices, glutamate evoked a rapid Ca^2+^ loading in the Purkinje neuron dendrites and somata (Fig. 1a–c, g, h, k, l; Movie S1). Although glutamate superfusion was maintained for 5 min, glutamate-induced Ca^2+^ loading was attenuated after ~ 2 min (Fig. 1g, h, l; Movie S1). This likely reflects reduction in Ca^2+^ influx over time and Ca^2+^ clearance. The average area under the curve (AUC) for the Ca^2+^ response during the initial 2 min of glutamate superfusion was significantly different between WT and *Trpc3*^*KO*^ somata (*p* = 0.0012; unpaired *t* test) (WT = 15.39 ± 1.78 Δ*F*/*F*_0_ s, *n* = 3 slices, 5–15 neurons per slice; 3 mice; *Trpc3*^*KO*^ = 6.55 ± 0.84 Δ*F*/*F*_0_ s, *n* = 6 slices, 3–10 neurons per slice; 5 mice) (Fig. 1k). This differential is consistent with Ca^2+^ entry via the mGluR1-TRPC3 pathway contributing ~ 57% of the total Ca^2+^ burden with sustained glutamate. The prominence of mGluR1-*Trpc3*-mediated Ca^2+^ entry was even greater in the dendrites (~ 67%) (Fig. 1d–f, l; Movie S2 shows minimal Ca^2+^ loading in *Trpc3*^*KO*^ dendrites). Whole field ROIs integrating the glutamate-induced Ca^2+^ dynamic showed that the TRPC3-mediated Ca^2+^ loading in the molecular layer predominated. The average AUC for the whole field Ca^2+^ response during the initial 2 min of glutamate superfusion was significantly different between WT and *Trpc3*^*KO*^ whole field ROIs (*p* = 0.009; unpaired *t* test) (WT = 2.36 ± 0.73 Δ*F*/*F*_0_ s, *n* = 3 slices, 3 mice; *Trpc3*^*KO*^ = 0.78 ± 0.59 Δ*F*/*F*_0_ s, *n* = 6 slices, 5 mice) (Fig. 1l).

**Fig. 2:**
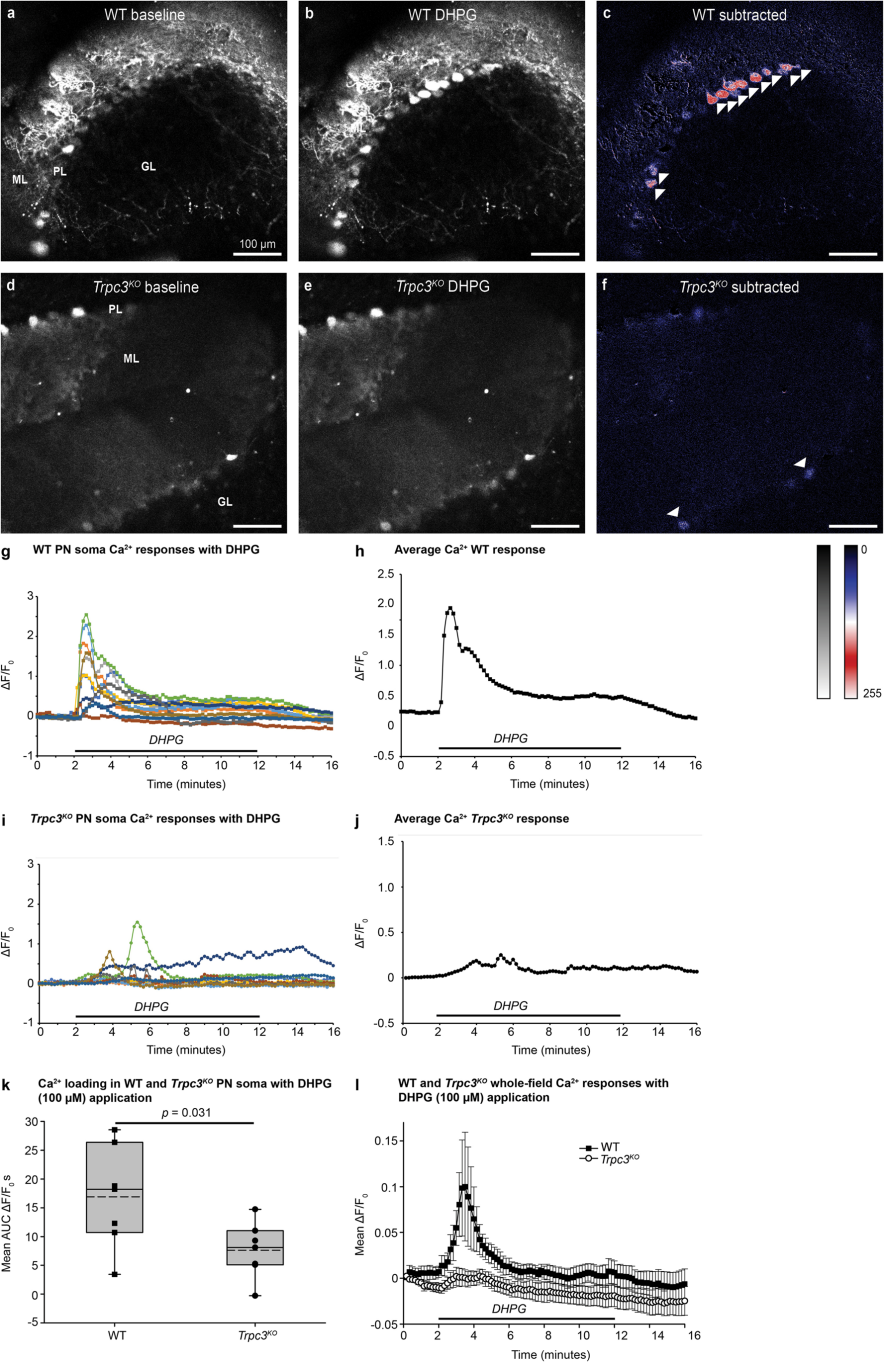
Ca^2+^ loading in Purkinje neurons evoked by application of the class I mGluR-selective agonist DHPG from WT or *Trpc3*^*KO*^ adult mouse cerebellar brain slices following AAV-GCaMP5g-transfection at P3. **a** Baseline. **b** Peak fluorescence with DHPG. **c** Ca^2+^ loading (subtraction of image **b**–**a**). This indicates that a significant proportion of the Ca^2+^ loading with glutamate is via this pathway. Application of DHPG to the *Trpc3*^*KO*^ tissue (**d**–**f**) resolved the independent Ca^2+^ store activation via the mGluR1-IP_3_R pathway. This was confined to the PL somata. **d** Baseline. **e** Peak intensity with DHPG. **f** Subtracted Ca^2+^ difference (**e**–**d**). There was no GCaMP5g signal in the granule cell layer (GL) in either the WT or *Trpc3*^*KO*^ slices. DHPG mGluR agonist-induced Ca^2+^ loading in Purkinje neurons (PN) in adult mouse WT and *Trpc3*^*KO*^ cerebellar brain slices. Kinetics of Ca^2+^ loading in Purkinje neurons (PN) from adult mouse WT and *Trpc3*^*KO*^ cerebellar brain slices utilizing GCaMP5g reporter with bath application of 100 μM DHPG. **g**, **h** WT individual and averaged Purkinje neuron soma Ca^2+^ signals in a representative brain slice. Adaptation occurs within the DHPG superfusion. **i**, **j** Limited *Trpc3*^*KO*^ individual and average Purkinje neuron soma responses during the DHPG presentation. **k** Comparison of the average area under the curve (AUC, 10 min) for PN somata responses across brain slices for WT (*n* = 3) and *Trpc3*^*KO*^ (*n* = 6). Box plots reflect 25% and 75% quartiles, with data overlay. Dashed lines show mean values; solid lines show the median. (*t* test). **l** Average DHPG-induced Ca^2+^ loading in WT and *Trpc3*^*KO*^ brain slices for whole field of view (reflecting PN dendritic Ca^2+^ signals in the molecular layer). Residual signal in the *Trpc3*^*KO*^ brain slices likely reflects Ca^2+^ store release. The difference reflects the significance of Ca^2+^ entry via the mGluR1-TRPC3 pathway. Data shown as mean ± SEM. Ca^2+^ fluorescence following AAV-GCaMP5g PN transfection to these adult mice at post-natal day 3. These brain slices correspond to those shown in Fig. 2 for initial glutamate treatment

To assess more specifically the mGluR-TRPC3-mediated Ca^2+^ loading in the WT brain slices, we superfused the mGluR1-selective agonist DHPG (Fig. 2a–c, g, h, k, l; Movie S3). As for glutamate application, DHPG elicited peak Ca^2+^ loading in the somata in the first 2 min, clearing to a lower sustained plateau for the remainder of the DHPG presentation (8 min, Fig. 2g, h). Dendritic Ca^2+^ loading was again dominant. Given that TRPC3 is the exclusive ion channel effector of mouse Purkinje neuron mGluR1 [24], it would be expected that application of DHPG to the *Trpc3*^*KO*^ cerebellar brain slices would result in a negligible Ca^2+^ response outside of transient store release. This was confirmed (Fig. 2d–f, i, j; Movie S4). The residual Ca^2+^ signal was confined largely to somata and likely arose from IP_3_-gated Ca^2+^ stores (Fig. S1). The average AUC for somata in WT slices over the 10-min DHPG superfusion was 16.92 ± 3.35 Δ*F*/*F*_0_ s (*n* = 7 slices, 4–15 neurons per slice; *n* = 6 mice), compared to *Trpc3*^*KO*^; 7.60 ± 1.83 Δ*F*/*F*_0_ s (*n* = 7 slices, 3–11 neurons per slice; *n* = 4 mice) (*p* = 0.031, two-tailed *t* test) (Fig. 2k). Similarly, whole field ROI integration (predominantly molecular layer signal) also showed significant differences, reflecting the prominent TRPC3-based Ca^2+^ entry in the WT Purkinje neuron dendrites (Fig. 2l). The average AUC for whole field ROI WT slices over a 10-min DHPG application = 1.18 ± 1.40 Δ*F*/*F*_0_ s (*n* = 7 slices; *n* = 6 mice), compared to *Trpc3*^*KO*^ = −0.66 ± 1.37 Δ*F*/*F*_0_ s (*n* = 7 slices; *n* = 4 mice) (*p* = 0.030, unpaired *t* test).

### TRPC3 Channels in Purkinje Neurons Contribute to Dendritic Excitotoxicity with Sustained Glutamate Exposure

**Fig. 3:**
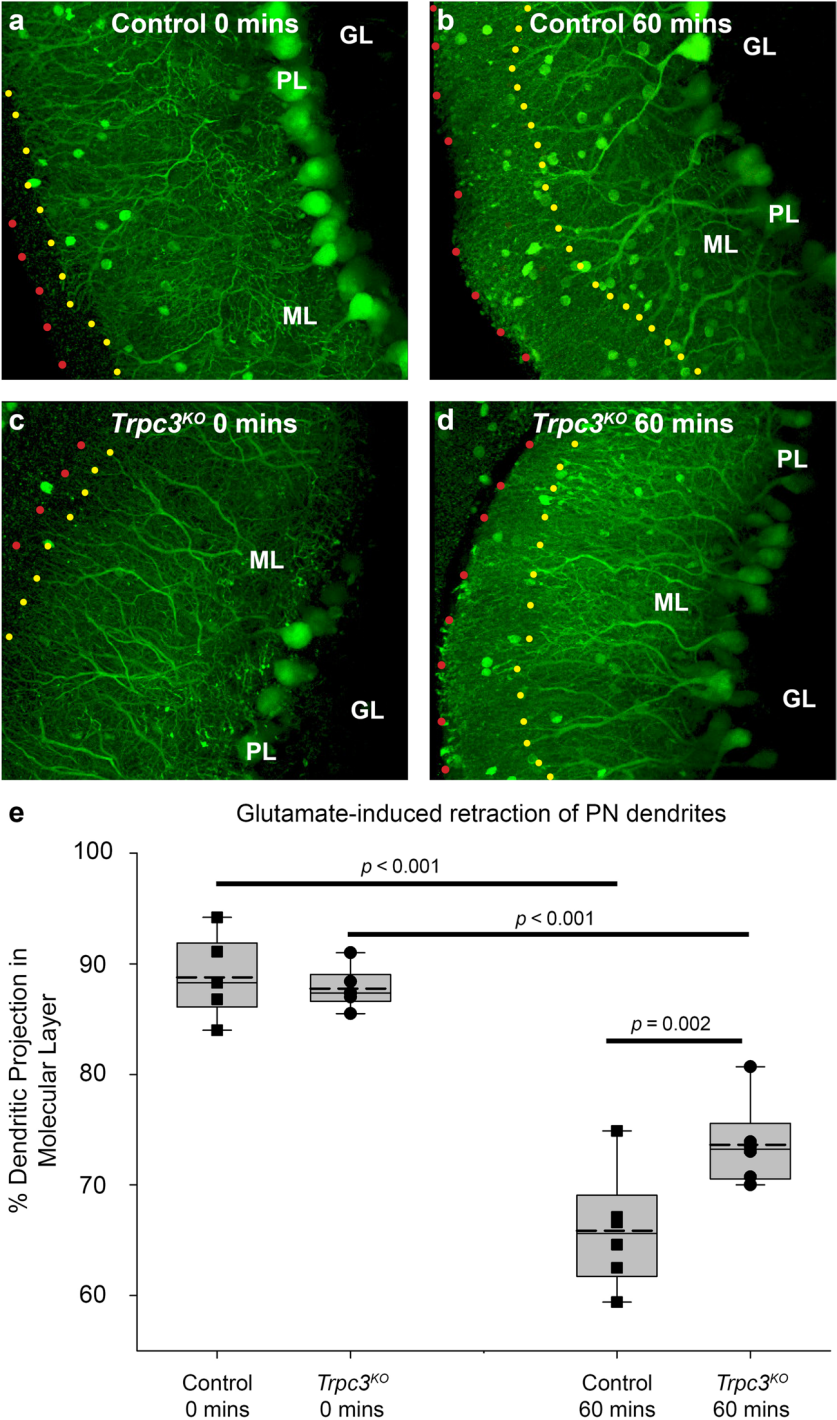
Glutamate exposure to *GAD67-GFP*^*+*^*-Trpc3*^*KO*^ cerebellar brain slices induced significantly less retraction of Purkinje neuron dendrites within the molecular layer compared with (control) *GAD67-GFP*^*+*^ brain slices. Representative images of parasagittal brain slices incubated with or without 1-mM glutamate for 60 min. Dotted overlay lines in **a**–**d** delineate the edge of the ML (red) and edge of the Purkinje neuron dendritic projections (yellow). **a** Control at 0 min. **b** Control at 60 min. **c** *Trpc3*^*KO*^ at 0 min. **d** *Trpc3*^*KO*^ at 60 min. **e** Retraction of Purkinje neuron dendritic projection within the molecular layer, calculated as the average length of the furthest projecting dendritic branch as a percentage of the molecular layer thickness. Box plots reflect 25% and 75% quartiles, with data overlay. Dashed lines show mean values; solid lines show the median. Each data point is the average of 16 measurements (4 dendrites measured for each of 4 randomized regions) from one brain slice per mouse per time point. The *Trpc3*^*KO*^ mice show significantly less dendrite retraction at 60-min glutamate exposure compared to the control. (two-way ANOVA, Holm-Sidak post hoc, *n* = 6 per genotype)

Given the prominence of mGluR1-*Trpc3* channel-mediated Ca^2+^ entry to the cerebellar molecular layer established by the Ca^2+^ imaging studies, the contribution of this pathway to glutamate-mediated excitotoxicity was assessed by comparing the effects of sustained glutamate stimulation in acute adult mouse parasagittal cerebellar brain slices from *GAD67-GFP*^*+*^ and *GAD67-GFP*^*+*^-*Trpc3*^*KO*^ mice. The GAD67-GFP reporter enabled high resolution imaging of the GABAergic Purkinje neuron dendrites within the molecular layer, enabling readout of necrosis. The cerebellar slices (randomized and blinded to genotype) were incubated in 1 mM glutamate in aCSF for 60 min at 31 °C to model glutamate excitotoxicity. Morphological degradation of the Purkinje neuron dendrites in the molecular layer was determined by measuring the retraction of the outer boundary of the dendrites in the molecular layer. The average baseline dendritic projections for *GAD67-GFP*^*+*^ and *GAD67-GFP*^*+*^-*Trpc3*^*KO*^ Purkinje neuron dendrites within the molecular layer were 88.80% ± 1.44% and 87.7% ± 0.75% respectively after 60 min of baseline incubation in aCSF (time 0) (*p* > 0.05; two-way ANOVA; *n* = 6 slices per time, average of 4 randomized regions/slice, for each genotype); imaged post-fixation. In separate brain slices incubated in glutamate for 60 min, the average projection length of the *GAD67-GFP*^*+*^ Purkinje neuron dendrites was reduced to 65.9% ± 2.15% of the span of the molecular layer (*p* < 0.001 compared to *t* = 0 *GAD67-GFP*^*+*^, two-way ANOVA, Holm-Sidak post hoc; *n* = 6 slices per time, average of 4 randomized regions/slice) (Fig. 3). In comparison, the Purkinje neuron dendrites in the *GAD67-GFP*^*+*^*-Trpc3*^*KO*^ brain slices following 60 min of glutamate treatment spanned 73.6% ± 1.55% of the molecular layer (*p* < 0.001, compared to *t* = 0 *GAD67-GFP*^*+*^*-Trpc3*^*KO*^; two-way ANOVA, Holm-Sidak post hoc; *n* = 6 slices, 4 regions/slice). The ~12% less retraction of the dendrites in the *GAD67-GFP*^*+*^*-Trpc3*^*KO*^ brain slices reflects significantly less damage post-excitotoxicity (*p* = 0.002, two-way ANOVA, Holm-Sidak post hoc). Associated with this quantitative analysis, the *GAD67-GFP*^*+*^*-Trpc3*^*KO*^ brain slices had subjectively less swelling of the primary dendrites and showed retention of fine spine structure (compare Fig. 3b, d). These was no significant effect of glutamate treatment on the surface area of the PN soma from WT or *Trpc3*^*KO*^ *GAD67-GFP*^*+*^ mouse brain slices (Fig. S3). This study indicates that block of the mGluR1-TRPC3 Ca^2+^ entry pathway confers neuroprotection from glutamate-mediated Purkinje neuron cell death.

### *Trpc3* and *Trpc1,3,6,7* Knockout Mice Exhibit Neuroprotection Following Focal Ischemia

Given the significant reduction in Ca^2+^ burden and dendritic glutamatergic excitotoxicity observed in the *Trpc3*^*KO*^ Purkinje neurons in vitro, the contribution of expression of *Trpc3* and *Trpc1*, *Trpc3*, *Trpc6*, and *Trpc7* genes in combination (in a *Trpc1/3/6/7* quadruple knockout (*Trpc*^*QKO*^)) was assessed in vivo using the photothrombotic model of focal ischemia. Infarcts were produced in both the cerebral and cerebellar brain regions simultaneously using dual light guides, across WT, *Trpc3*^*KO*^, and *Trpc*^*QKO*^ mice, with the investigator blinded to genotype.

**Fig. 4:**
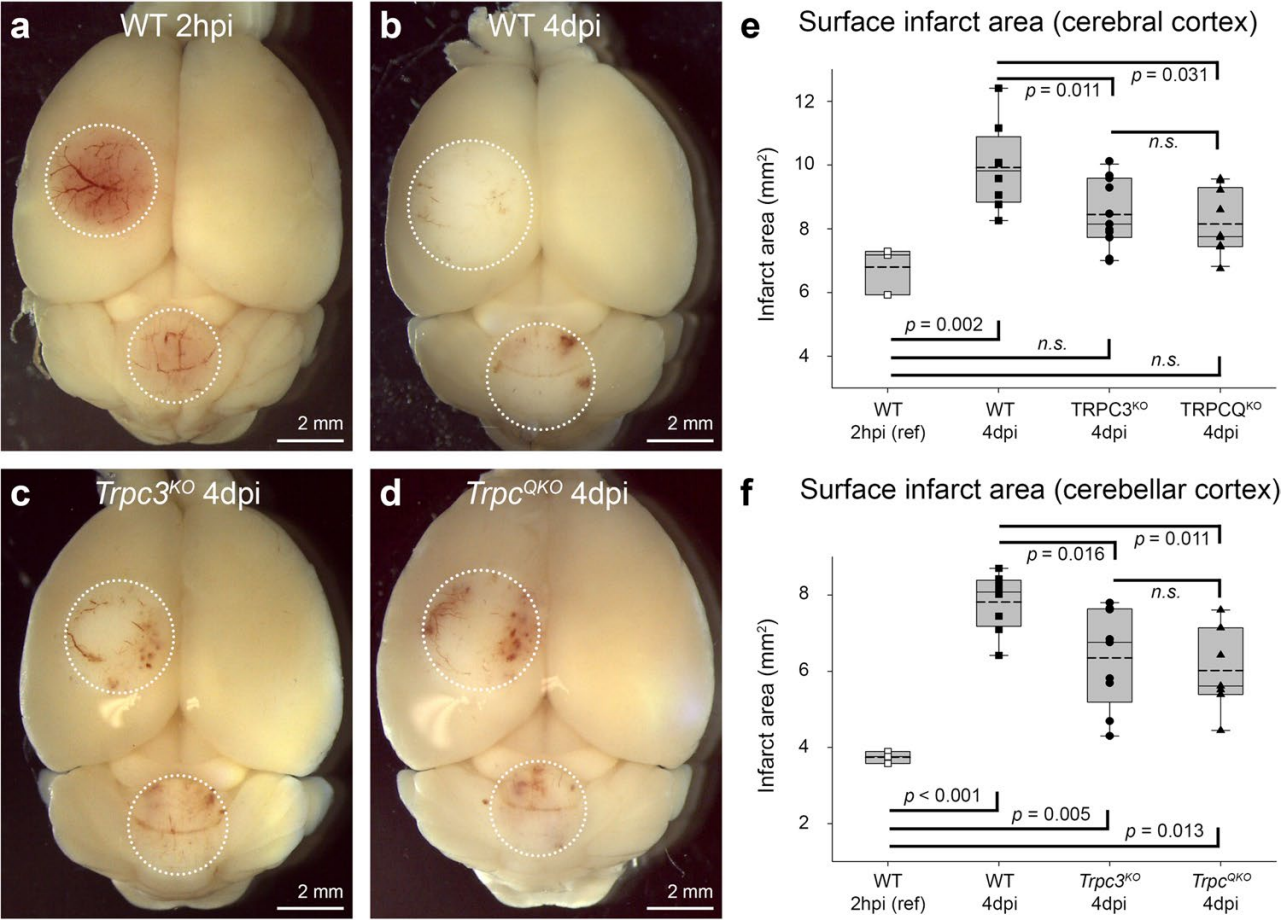
Gross area of injury on the surface of the cerebral cortex and cerebellar cortex arising from dual photothrombotic lesions in WT, *Trpc3*^*KO*^, and *Trpc*^*QKO*^ mouse brains. Representative images of **a** WT at 2-h post-injury (2 hpi). **b** WT 4-day post-injury (4 dpi). **c** *Trpc3*^*KO*^ 4 dpi. **d** *Trpc*^*QKO*^ 4 dpi. White circles demarcate the ischemic/infarct region (blinded with respect to genotype). Blood clots can be seen within the ischemic region. The surface infarcts at 4 dpi in the *Trpc3*^*KO*^ and *Trpc*^*QKO*^ mice were smaller than those of WT mice in both **e** cerebral cortex and **f** cerebellar cortex (one-way ANOVA, Holm-Sidak post hoc; cerebrum *n* = 8, WT; *n* = 11, *Trpc3*^*KO*^, *n* = 10, *Trpc*^*QKO*^; for cerebellum *n* = 8, WT; *n* = 9, *Trpc3*^*KO*^, and *n* = 7, *Trpc*^*QKO*^). The WT 2 hpi (*n* = 3) data reflects the irrecoverable primary photothrombotic infarct caused by platelet aggregation in the microvasculature; 4 dpi data reflects secondary expansion of the brain injury. Box plots reflect 25% and 75% quartiles, with data overlay. Dashed lines show mean values; solid lines show the median

The size of the primary infarct (irreversible photothrombotic microvascular blockage) was established on the intact surface of the brain in a subgroup of WT mice at 2-h post-injury (2 hpi; 6.80 ± 0.44 mm^2^; *n* = 3). In separate groups, the expansion of secondary brain injury was assessed at 4-day post-injury (4 dpi) across the genotypes. In the WT cerebral cortex, this secondary brain injury expansion was evident as a 46% increase in the area of infarct on the brain surface (9.92 ± 0.48 mm^2^ at 4 dpi (*n* = 8); *p* = 0.002 compared with 2 hpi, one-way ANOVA, Holm-Sidak post hoc). In comparison, the average surface area of the infarct at 4 dpi in *Trpc3*^*KO*^ was 8.45 ± 0.32 mm^2^ (*n* = 11) and for *Trpc*^*QKO*^ was 8.15 ± 0.32 mm^2^ (*n* = 10). This indicated a secondary infarct expansion of ~ half that seen in the WT between 2 hpi and 4 dpi: 24% for *Trpc3*^*KO*^; 20% for *Trpc*^*QKO*^. The overall infarct size at 4 dpi in the *Trpc3*^*KO*^ and *Trpc*^*QKO*^ mice was not significantly different from the primary infarct measured at 2 hpi in WT slices (*p* > 0.05, ANOVA). The 4 dpi comparisons indicated significant overall reduction in infarct surface area compared with WT for both TRPC null genotypes (*p* = 0.031 *Trpc3*^*KO*^ vs WT; *p* = 0.011 *Trpc*^*QKO*^ vs WT; one-way ANOVA, Holm-Sidak post hoc; Fig. 4a–e).

In the WT cerebellum, the primary infarct area on the brain surface (2 hpi) averaged 3.74 ± 0.09 mm^2^ (*n* = 3), expanding by 109% to 7.82 ± 0.27 mm^2^ in the 4 dpi WT group (*n* = 8) (*p* < 0.001; one-way ANOVA, Holm-Sidak post hoc). In comparison with the WT 2 hpi primary infarct reference, the secondary brain injury expansion at 4 dpi in *Trpc3*^*KO*^ mice was 70% (to 6.35 ± 0.43 mm^2^; *n* = 9; *p* = 0.005), and in *Trpc*^*QKO*^ mice, it was 61% (6.02 ± 0.42 mm^2^; *n* = 7; *p* = 0.013). The 4-dpi brain surface infarct areas for both the *Trpc3*^*KO*^ and *Trpc*^*QKO*^ were significantly less than the 4-dpi WT infarct area (*p* = 0.016 and *p* = 0.011 respectively) (Fig. 4a–d, f). Overall, the neuroprotection from secondary injury conferred to the cerebellum by knockout TRPC channels was ~40–50% (there was no significant difference between infarct surface areas in *Trpc3*^*KO*^ vs *Trpc*^*QKO*^).

**Fig. 5:**
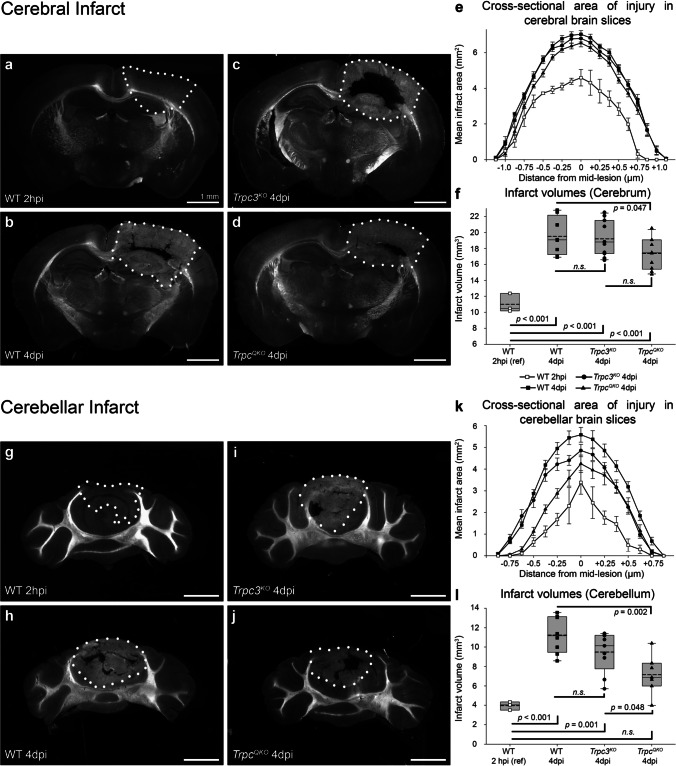
Mice null for *Trpc1,3,6,7* ion channel subunit(s) (*Trpc*^*QKO*^) showed significant neuroprotection from secondary infarct volume expansion over 4 days following photothrombotic lesion in both cerebrum and cerebellum. Infarct volumes were calculated by serial reconstruction from coronal sections imaged using darkfield microscopy (50-μm cryosections). Representative darkfield images of mid-lesion sections from the cerebral and cerebellar regions respectively of **a**, **g** WT mouse at 2-h post-injury (2 hpi). **b**, **h** WT at 4-day post-injury (4 dpi). **c**, **i** *Trpc3*^*KO*^ mouse at 4 dpi. **d**, **j** *Trpc*^*QKO*^ mouse at 4 dpi. The infarcted brain tissue (demarcated by white dots) in all these sections has an opaque appearance compared to the translucent appearance of the healthy surrounding tissue. **e** Cerebral infarct cross-sectional area of individual sections (every 5th section shown). **f** Cerebral infarct volume (*t* test comparison between WT and *Trpc*^*QKO*^; Kruskal-Wallis one-way ranked ANOVA, 2-h post-injury (2 hpi) vs 4-day post-injury (4 dpi) groups; *n* = 3 WT 2 hpi; *n* = 8 WT 4 dpi; *n* = 11 *Trpc3*^*KO*^ 4 dpi; *n* = 10 *Trpc*^*QKO*^ 4 dpi). **k** Cerebellar infarct cross-sectional area **l** Cerebellar infarct volume (one-way ANOVA, Holm-sidak post-hoc comparisons; *n* = 3 WT 2 hpi; *n* = 8 WT 4 dpi; *n* = 9 *Trpc3*^*KO*^ 4 dpi; *n* = 7 *Trpc*^*QKO*^ 4 dpi). The boxplots show the 25% and 75% interquartile boundaries, SEM, median (solid line), and the mean (dashed line) overlaid with the individual infarct volumes. WT 2 hpi data reflect primary infarct volume arising from thrombotic occlusion of the microvasculature

Following imaging of the brain surface infarcts, darkfield imaging of the serial coronal cryosections was used to delineate the infarct cross-sectional areas along the rostro-caudal axis, for infarct volume reconstruction in both cerebral and cerebellar brain regions (Fig. 5). Blinded darkfield imaging of the brain sections was adopted, as the mapping of the injury boundary delineated by the differential in optical diffraction was deemed superior to conventional histology and minimized processing artifacts (Fig. S4). The serial sectioning demonstrated a rostro-caudal expansion of the cerebral cortex infarct in WT from ~ 4.25 mm at 2 hpi to 5 mm at 4 dpi. In the cerebellum, the injury expanded from a rostro-caudal average of 2.75 mm at 2 hpi to 3.55 mm at 4 dpi. The injury area within the cryosections within the infarct zone increased significantly over the 4 days for WT and the two *Trpc* KO groups, but there was significantly less brain damage for the *Trpc*^*QKO*^ genotype across both cerebral and cerebellar infarcts. Average 4-dpi cerebral cortex infarct volumes were WT 4 dpi = 19.53 ± 0.83 mm^3^; *Trpc3*^*KO*^ = 19.20 ± 0.65 mm^3^; *Trpc*^*QKO*^ = 17.36 ± 0.61 mm^3^ (*p* = 0.047; *t* test comparison with WT 4 dpi). Cerebellar 4-dpi infarct volumes were WT 4 dpi = 11.25 ± 0.67 mm^3^; *Trpc3*^*KO*^ = 9.48 ± 0.69 mm^3^; *Trpc*^*QKO*^ = 7.16 ± 0.76 mm^3^ (*p* = 0.002; *t* test comparison with WT 4 dpi). Given a primary injury volume (2 hpi) of 11.00 ± 0.68 mm^3^ for the cerebrum, this represents a secondary expansion of ~76% in the WT versus 58% for the *Trpc*^*QKO*^; for the cerebellum, the secondary injury expansion was 184% in the WT against 88% for the *Trpc*^*QKO*^ (relative to WT 2-hpi infarct volume = 3.96 ± 0.24 mm^3^).

## Discussion

This study establishes a primary role for G_αq_-coupled TRPC ion channels in excitotoxicity driving secondary expansion of brain injury. The cerebellar brain slice Ca^2+^ imaging studies showed that sustained activation of the class I mGluR by both glutamate and the selective agonist DHPG produced equivalent Ca^2+^ loading of the Purkinje neuron dendrites in the molecular layer of WT mice brain slices, that was largely suppressed in the *Trpc3*^*KO*^ brain slices. Ca^2+^ loading of the Purkinje neuron somata also exhibited a major mGluR1-*Trpc*3 component. These neurons were also protected from acute glutamatergic excitotoxicity in *Trpc3*^*KO*^ brain slices, while with focal ischemic brain injury in vivo, the knockout of signaling involving additional G_αq_-coupled TRPC channel subunits was needed to limit the expansion of the penumbra in the cerebral and cerebellar cortex regions. These data indicate the G_αq_-coupled TRPC Ca^2+^ entry pathway is a major contributor to the expansion of brain injury in stroke, and likely contributes to related glutamate triggered neuropathologies including TBI and epilepsy.

The exclusive coupling of mGluR1 signaling to TRPC3 channels was established in cerebellar Purkinje neurons using the *Trpc3*^*KO*^ mouse model [24]. The Purkinje neurons in these *Trpc3*^*KO*^ mice lack the slow EPSCs evident in WT neurons following brief electrical stimulation of the parallel fiber input. However, IP_3_R-gated Ca^2+^ store activation was still detected in these *Trpc3*^*KO*^ mice, reflecting mGluR1-mediated PLC_β_ activation of that parallel PiP_2_ hydrolysis product. That finding is consistent with dominance of Ca^2+^ signaling from intracellular stores over extracellular Ca^2+^ entry via TRPC3 channels during physiological stimulation. However, the current study indicates that with dysregulation of glutamate release in pathophysiological conditions, Ca^2+^ entry via the parallel DAG-mediated activation of the TRPC3 channels dominates. This may be particularly prominent in Purkinje neurons; alternative splicing of the *Trpc3* mRNA transcript leads to TPC3 channels with very high membrane Ca^2+^ conductance; equivalent to that of the ionotropic NMDA receptors [6]. This alternative splicing of *Trpc3* mRNA is evident in multiple brain regions across rodents and the human brain [25]. NMDA receptors are considered to be the principal driver of glutamate excitotoxicity across the brain, and have been a primary, albeit unsuccessful target for stoke and TBI therapeutics [34]. However NMDA receptor expression is low at the parallel fiber synapses on the Purkinje neuron dendrites [35], where the GluR2 low-Ca^2+^ permeability AMPA receptor is the dominant ionotropic receptor [36], and mGluR1 is the predominant Purkinje neuron glutamatergic GPCR receptor assembled as a mGluR1a/1b dimer in the dendritic spines [37]. The present data demonstrate mGluR1-TRPC3 to be the dominant Ca^2+^ entry pathway in these neurons, with caveats around potential secondary activation of voltage-gated Ca^2+^ channels via TRPC3-mediated depolarization, and Ca^2+^-induced Ca^2+^ release mechanisms, which are nevertheless both tied to the primary class I mGluR-TRPC3 pathway [9]. Since in the *Trpc3*^*KO*^ both glutamate (activating ionotropic and metabotropic glutamate receptors) and DHPG (activating class I mGluR) exhibited equivalent residual ~ 30% Ca^2+^ loading of the somata, this most likely reflects activation of the parallel PCL_β_-IP_3_R Ca^2+^ store release pathway. As such, this indicates that in the cerebellar Purkinje neurons, virtually, all glutamate-mediated Ca^2+^ entry is via the mGluR1-TRPC3 effector channel pathway. This has clear implications for the vulnerability of these neurons to excitotoxicity, which likely contributes to the accelerated secondary brain injury in the cerebellar cortex compared with the cerebral cortex [32] and the exacerbated pathology associated with posterior circulation stroke [38–40]. Follow-on studies utilizing this photothrombotic model of focal ischemia could evaluate neuroprotection of motor function, while controlling for a mild gait-related cerebellar ataxia phenotype identified in the *Trpc3*^*KO*^ likely attributable to the Purkinje neurons [24]. The enhanced neuroprotection achieved in the *Trpc*^*QKO*^ mouse model is consistent with the broad distribution of the related PLC_β_-activated TRPC6 and TRPC7 subunits, alongside TRPC3 subunits throughout the brain, including the cerebellu﻿m [41] (https://mouse.brain-map.org/).

The TRPC channels most readily activated by the G_αq_-PCL_β_-PIP_2_-DAG pathway include TRPC3,6,7 subunits [11] with broad expression across the brain [6, 24, 25, 42]. Their expression patterns show strong overlap with expression patterns of G_αq_-type GPCRs, activated by both glutamatergic and non-glutamatergic signaling [20]. Activation of G_αq_-type GPRCs with brain injury, and by implication their coupling to TRPC Ca^2+^-entry channels, encompass a range of additional neurotransmitters and neuromodulators in neurons, astrocytes, microglia, microvascular endothelial cells, and platelets. Prominent among these G_αq_-type GPCRs are muscarinic acetylcholine receptors (M3 mAChR) [43]; P2Y_1_ purinergic receptors activated by ischemia-induced ATP release [44, 45]; protease-activated receptors (PAR1, PAR4) activated by thrombin [46–49]; and the endothelin receptor (ET_B_) [50, 51], all of which are implicated in stoke pathology.

This broad capacity for loss of homeostatic regulation of TRPC channels to drive brain injury pathophysiology across neurons and glia is supported by in vitro and in vivo studies that complement the current findings. *Trpc3* expression is the prominent subtype in cortical astrocytes, and in a mouse penetrating cortical stab wound model of TBI, *Trpc3*^*KO*^ mice had reduced astrogliosis based on GFAP-positive activated astrocyte immunofluorescence in the injury zone [14]. Multi-gene deletion knockout mouse models have provided a highly selective tool for evaluating the contribution of TRPC channels in ischemic brain injury. Chen et al. [26] used a MCAO model with reperfusion after 90 min to compare lesion volumes at 24-h post-infarct in *Trpc3,6,7* KO mice versus wildtype mice. The absence of these TRPC channels reduced infarct volumes by 50%, improved neurological deficit scores, and reduced molecular markers of apoptosis. Recently, the *Trpc1,4,5,6* quadruple KO mouse has also been assessed in the MCAO-reperfusion stroke model, with comparable (~50%) reduction in infarct volumes and again improved neurological deficit scores [8]. These findings are complemented by the present photothrombotic infarct study with the *Trpc1,3,6,7*^*QKO*^ mouse line, where secondary expansion of focal ischemic brain injury over 4 days, from the primary (irrecoverable) infarct determined at 2 h provided 24–52% neuroprotection from the secondary cerebral and cerebellar cortex brain injuries respectively compared with the wildtype (Fig. 5f, l).

In the present Ca^2+^ imaging experiments, Ca^2+^ loading with sustained presentation of glutamate or DHPG in WT brain slices showed a characteristic “peak and plateau” profile, where peak Ca^2+^ levels from individual Purkinje neurons, or ROIs covering substantial regions of the cerebellar molecular layer, were sustained for ~ 3 min before reducing to a steady-state level to the end of the superfusion period (5–10 min). This adaptation of TRPC channel-mediated Ca^2+^ loading may in part represent caveolin-mediated internalization of the mGluR1α [52]. It is also likely to reflect the inhibition of TRPC channels with elevation of cytosolic Ca^2+^. As demonstrated with heterologous expression of TRPC3b and TRPC3c isoforms in HEK293 cells, Ca^2+^ applied to inside-out patches inhibits channel gating [6]. This adaptation involves both the cytosolic CIRB (calmodulin-IP_3_R binding) motif conserved across TRPC channels [53], and non-CIRB mechanisms. The latter is evident from the TRPC3c adaptation, where this splice variant lacks exon 9, that encodes part of the calmodulin binding element of the CIRB domain [6, 25], and exhibits five times the macroscopic conductance of the unspliced TRPC3 isoform. As a corollary, in HEK293 cells, co-expression of mGluR1 drives similar TRPC3-mediated Ca^2+^ entry via DHPG, as carbachol-mediated endogenous M3 mAChR activation [6]; demonstrating the commonality of the GPCR-G_αq/ll_-PLC_β_ activation pathway. Similar adaptation is evident in TRPC6 and TRPC7 homomeric channels [54, 55]. Such adaptation may limit somewhat the Ca^2+^ loading during pathological sustained glutamate exposure, and loss of this adaptation in cells expressing the TRPC3c variant (e.g., Purkinje neurons) confers vulnerability to excitotoxic damage. However, clearly multiple TRPC subunits can contribute to Ca^2+^ homeostasis in many cell types. Indeed, given the multiplicity of neurotransmitters and neuromodulators within the same neural circuits, stroke-induced ischemia or trauma will inevitably release a cocktail of G_αq_ type GPCR agonists, with TRPC-mediated Ca^2+^ entry as a common effector endpoint with possible activation across multiple receptors, even in the same cell (as summarized in Fig. S1).

In the cerebellar brain slices from adult mice, expression of the GCaMP5G protein was only observed in the molecular and Purkinje neuron layers, with no expression in the granule cell layer. This could not have been promoter-related, as gene delivery into the cerebellum with the CAG promotor has previously shown transduction in the cerebellar granule cells [56]. Only a small percentage of the vector genome typically integrates into the host cell genome, whereas the majority remains extrachromosomal (episomal expression). Proliferating cells normally lose the unintegrated AAV vector [57, 58]. At P3, the age at which the AAV injections were performed in this study, Purkinje neurons were the only cerebellar cortical neurons in their post-mitotic stage, whereas the granule, basket, and stellate cells were still in the proliferating stage [59].

In conclusion, TRPC3 ion channels were found to mediate the majority of glutamate-mediated Ca^2+^ entry into cerebellar Purkinje neurons with sustained activation of the G_αq_-coupled mGluR1 receptor. More broadly, the diversity of TRPC3,6,7 subunit assembly into non-selective cation channels readily activated by G_αq_-PLC_β_-DAG second messenger signaling confers vulnerability to dysregulation of excitatory neurotransmitters, as evident from the greater neuroprotection against secondary injury expansion afforded by the *Trpc1*,*3*,*6*,*7* quadruple knockout mouse model over the *Trpc3*^*KO*^. Overall, the demonstrated contribution of the G_αq_-type GPCR-TRPC channel effector pathway to secondary brain injury expansion in the hours and days following primary injury is indicative of the dominance of Ca^2+^ burden mediated by TRPC channel signaling, and may be emblematic of a more systematic impact of this signal transduction mechanism across a breadth of neuronal and glial cell pathophysiology. These findings consolidate the case for clinical evaluation of TRPC channel blockers for neuroprotection from secondary brain injury in stroke, traumatic brain injury, and epilepsy, areas of the highest unmet need for therapeutics. The TRPC Ca^2+^ entry channels linked to G_αq_-type GPCRs represent a drug target at arms-length from the primary neurotransmitter receptors of the brain, where adverse psychophysical reactions have long thwarted clinical translation of neuroprotective drug candidates.

### Supplementary Information

ESM 1 (PDF 1254 kb)

ESM 2 (MP4 92079 kb)

ESM 3 (MP4 99970 kb)

ESM 4 (MP4 161779 kb)

ESM 5 (MP4 153792 kb)
