# Supplementary material for: TRPC Channels Activated by G Protein-Coupled Receptors Drive Ca2+ Dysregulation Leading to Secondary Brain Injury in the Mouse Model
Source: Transl Stroke Res. 2023 Jul 18;15(4):844–58. doi: 10.1007/s12975-023-01173-1 (PMC11226524; doi:10.1007/s12975-023-01173-1)
Supplement: Supplementary file 1 — (PDF 1254 kb) [file 12975_2023_1173_MOESM1_ESM.pdf]

Supplementary Information
(Parmar *et al.*)

TRPC channels activated by G protein - coupled receptors drive Ca^{2+} dysregulation leading to secondary brain injury in the mouse model

Jasneet Parmar[1], Georg von Jonquieres[1], Nagarajesh Gorlamandala[1], Brandon Chung[1], Amanda J. Craig[1], Jeremy L. Pinyon[1], Lutz Birnbaumer[2,3], Matthias Klugmann[1], Andrew J. Moorhouse[1], John M. Power[1], Gary D. Housley[1*]

[1]Translational Neuroscience Facility and Department of Physiology, School of Biomedical Sciences, UNSW Sydney, Sydney, NSW 2052, Australia.
[2]Institute of Biomedical Research (BIOMED), Pontifical Catholic University of Argentina, Argentina, Av A Moreau de Justo 1300, C1107AFF Buenos Aires CABA Argentina.
[3] Laboratory of Signal Transduction, National Institute of Environmental Health Sciences, Research Triangle Park, North Carolina 27709, USA.

Gary D. Housley
Email: g.housley@unsw.edu.au

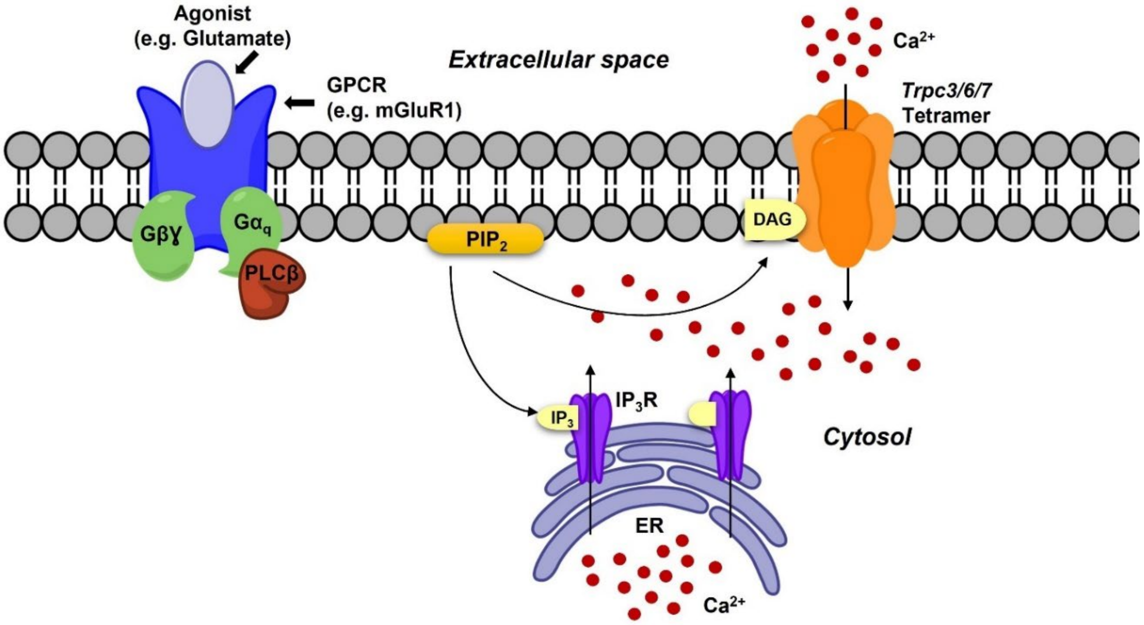

Fig. S1 Model of Ca^{2+} entry in the brain via TRPC non-selective cation channels coupled to $G\alpha_q$ – type G protein-coupled receptors (GPCRs). Channels assembled from TRPC3/6/7 subunits are readily activated by diacylglycerol (DAG) through phospholipase C_β (PLC_β) – mediated conversion of phosphoinositol bisphosphate (PIP_2). The schematic coupling to the class I metabotropic glutamate receptor mGluR1. This pathway is proposed here as a major contributor to neurodegenerative processes (excitotoxicity) following brain ischemia, due to sustained release of glutamate at synapses and by astrocytes, leading to excessive depolarization (including Na^+ and Ca^{2+} entry via the TRPC non-selective cation channel effector pathway; complementing ionotropic GluR activation), with sustained Ca^{2+} loading that is maintained beyond the transient depletion of Ca^{2+} stores via the complementary endoplasmic reticulum (ER) inositol trisphosphate receptors (IP_3R). This TRPC channel Ca^{2+} entry pathway will also be activated by the other $G\alpha_q$ protein – coupled receptors for neurotransmitters and modulators, such as acetylcholine (via the muscarinic acetylcholine receptor) and adenosine triphosphate (ATP – via P2Y receptors) across neurons, astrocytes, and microglia.

Table S1

Number and average ages of male and female mice across the three genotypes for the dual photothrombotic brain infarct model

	Number of males (average age in days)		Number of females (average age in days)	
	Cortex	Cerebellum	Cortex	Cerebellum
WT	5 (59)	6 (59)	3 (71)	2 (75)
Trpc3^{KO}	6 (70)	6 (70)	5 (74)	3 (69)
Trpc^{QKO}	5 (74)	4 (76)	5 (64)	3 (67)

Supplementary Information
(Parmar *et al.*)

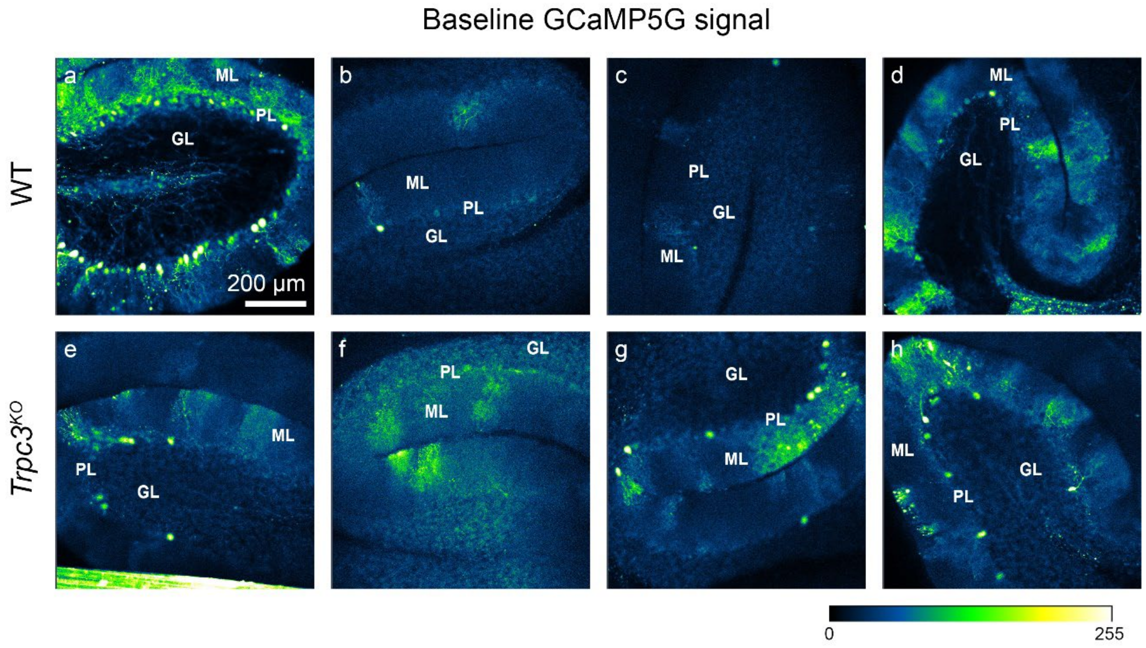

Fig. S2 Representative adult cerebellar brain slices from WT and *Trpc3*^{KO} mice showing baseline GCaMP5g fluorescence. This demonstrates the comparable distribution of signal across regions arising from AAV delivery when the mice were neonates (P3).

Supplementary Information
(Parmar *et al.*)

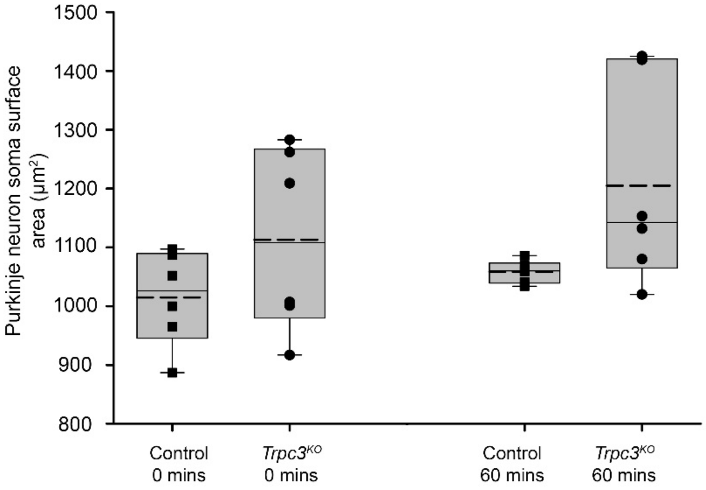

Fig. S3 Measurement of Purkinje neuron (PN) somata surface areas before and after treatment with glutamate (1 mM, 60 minutes). Box plots reflect 25% and 75% quartiles, with data overlay. Dashed lines show mean values; solid lines show the median. Each data point is the average neuron surface area of 3 – 4 neurons within each of four regions of interest from a cerebellar brain slice from one mouse. There were no significant differences across the treatment groups (p = 0.166; Kruskal-Wallis one way ANOVA on Ranks). Control is *GAD67-GFP*$^+$ mouse. *Trpc3*^{KO} is the *GAD67-GFP*$^+$-*Trpc3*^{KO} mouse. PN surface areas were determined from 3D reconstructions based on z stacks of images acquired using multiphoton laser scanning microscopy (see Methods).

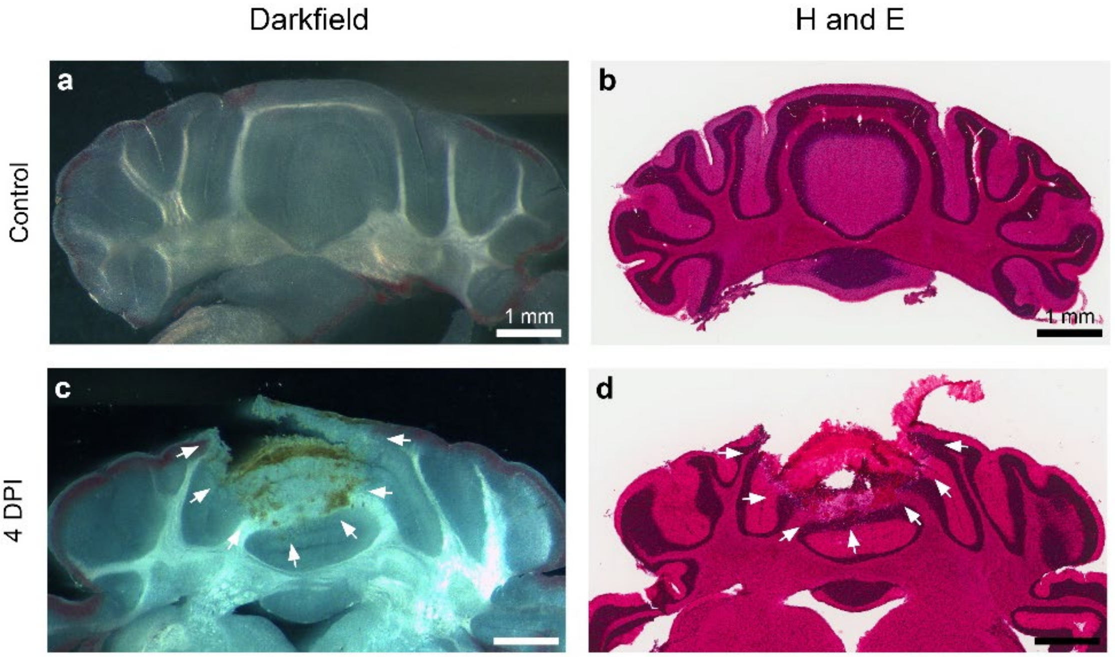

Fig. S4 Visualization of the photothrombotic infarct in mouse cerebellum. Comparison of darkfield images of unprocessed 50 µm mouse cerebellar cryosections, with re-imaging following hematoxylin and eosin (H&E) histology. a and b are images of a control cerebellar section from a sham- operated mouse (light exposure, but no intravenous Rose Bengal delivery), reflecting healthy tissue. c and d are respective darkfield and H&E images of a section from mid-infarct region four days post-injury. The darkfield image provides superior delineation of the injury boundary as a change in optical diffraction between the healthy tissue and the infarcted zone. Dark field imaging also minimized processing artifacts inherent to handling infarcted brain tissue.

Supplementary Information
(Parmar *et al.*)

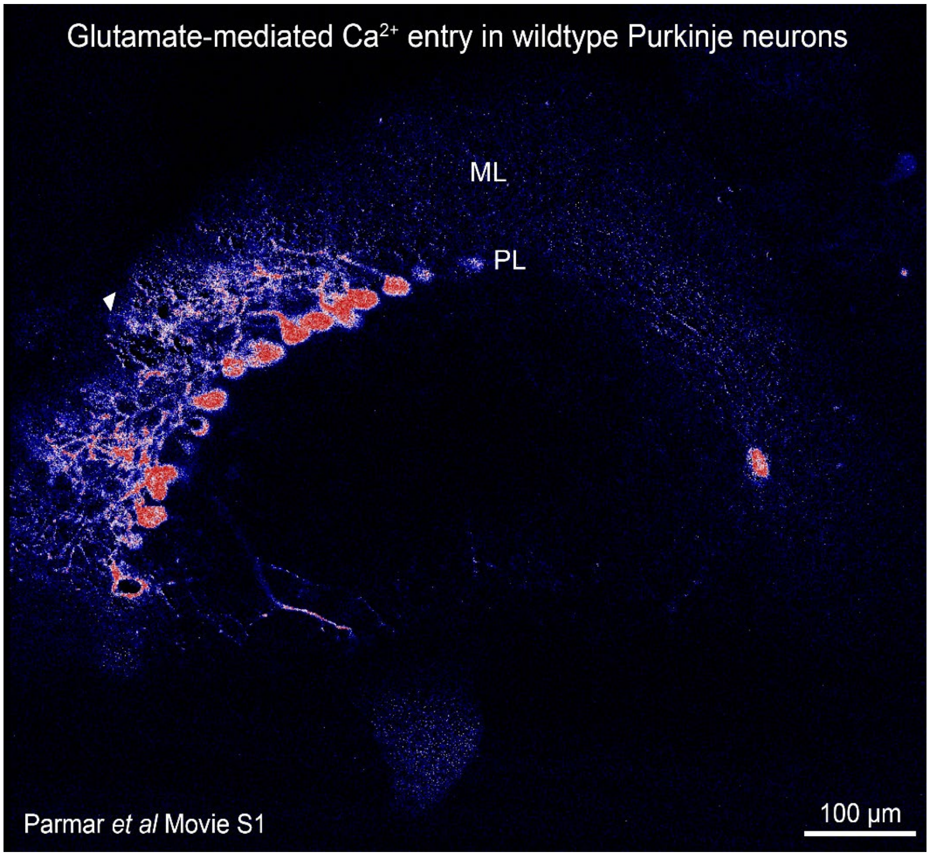

Movie S1 Ca^{2+} dynamics in a wildtype adult mouse cerebellar brain slice during 5 minutes of 4 mM glutamate application. Note the rapid loading of Ca^{2+} into the Purkinje neuron dendritic arbor and increased signal in the soma. The pronounced Ca^{2+} loading in the dendrites is attributable to metabotropic receptor (mGluR1) activation of TRPC3 ion channels (see Supplementary Figure 1). The frame rate was 6 / minute, with background subtraction of the baseline signal just prior to glutamate application. Transfection of the brain tissue with the AAV-GCaMP5g genetically encoded Ca^{2+} reporter vector was achieved *in vivo* by microinjection into the cerebellum at post-natal day 3, and brain slice preparation occurred at 8 weeks of age. – supporting Figs 1,2.

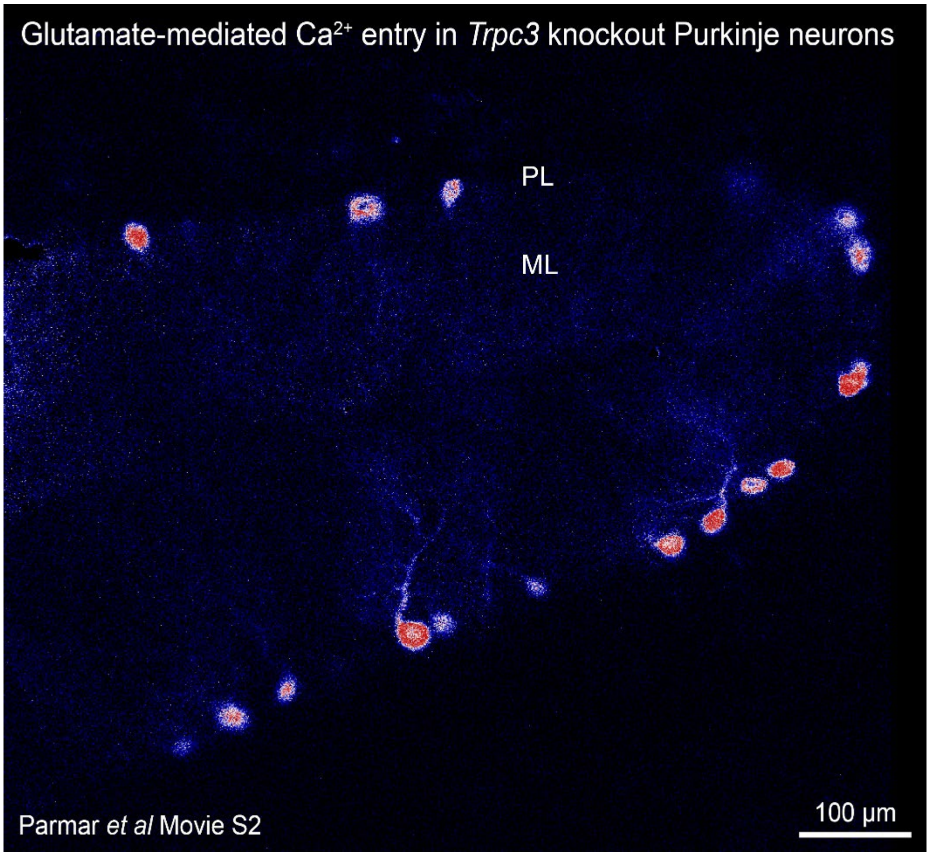

Movie S2 Attenuated Ca^{2+} dynamics in a TRPC3 knockout adult mouse cerebellar brain slice during 5 minutes of 4 mM glutamate application. Note the limited loading of Ca^{2+} into the Purkinje neuron dendritic arbor compared with the wildtype slice example (compare with Supplementary Video 1), while Ca^{2+} loading occurs in the soma; primarily attributable to direct activation of Ca^{2+} store-release via the metabotropic glutamate receptor (mGluR1) – phospholipase C – inositol trisphosphate pathway (see Supplementary Fig 1). The frame rate was 6 / minute, with background subtraction of the baseline signal just prior to glutamate application. Transfection of the brain tissue with the AAV-GCaMP5g genetically encoded Ca^{2+} reporter vector was achieved *in vivo* by microinjection into the cerebellum at post-natal day 3, and brain slice preparation occurred at 6 weeks of age. – supporting Figs 1,2.

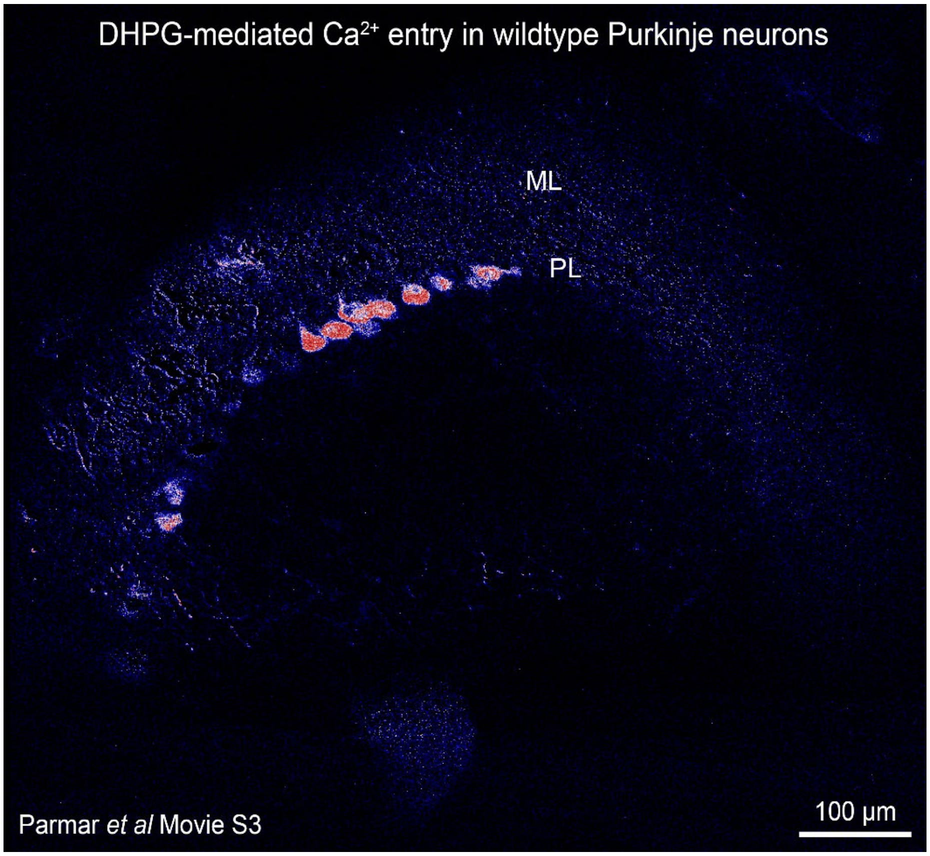

Movie S3 Ca^{2+} dynamics in a wildtype adult mouse cerebellar brain slice during 10 minutes of 100 µM DHPG (class I mGluR agonist) application directed to selectively activate the metabotropic glutamate receptor pathway. Ca^{2+} loading is sustained in the Purkinje neuron dendritic arbor. The frame rate was 6 / minute, with background subtraction of the baseline signal just prior to glutamate application. Transfection of the brain tissue with the AAV-GCaMP5g genetically encoded Ca^{2+} reporter vector was achieved *in vivo* by microinjection into the cerebellum at post-natal day 3, and brain slice preparation occurred at 8 weeks of age. – supporting Figs 1,3.

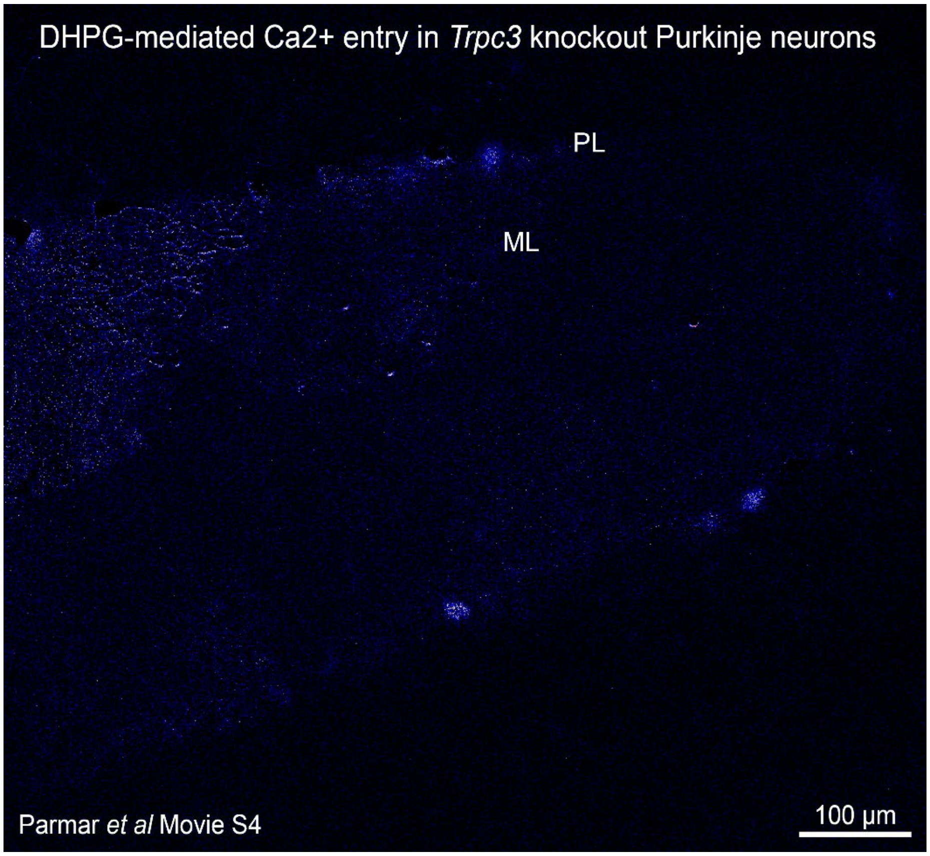

Movie S4 Ca^{2+} dynamics in a *Trpc3*^{KO} adult mouse cerebellar brain slice during 10 minutes of 100 µM DHPG (class I mGluR agonist) application directed to selectively activate the metabotropic glutamate receptor pathway. Ca^{2+} loading is minimal and largely confined to the Purkinje neuron somata. The frame rate was 6 / minute, with background subtraction of the baseline signal just prior to glutamate application. Transfection of the brain tissue with the AAV-GCaMP5g genetically encoded Ca^{2+} reporter vector was achieved *in vivo* by microinjection into the cerebellum at post-natal day 3, and brain slice preparation occurred at 6 weeks of age. – supporting Figs 1,3.
